# A workflow for low-cost automated image analysis of myxomycete spore numbers, size and shape

**DOI:** 10.7717/peerj.12471

**Published:** 2021-11-16

**Authors:** Jan Woyzichovski, Oleg Shchepin, Nikki Heherson Dagamac, Martin Schnittler

**Affiliations:** 1 Institute of Botany and Landscape Ecology, Greifswald University, Greifswald, Mecklenburg-Western Pomerania, Germany; 2 Laboratory of Systematics and Geography of Fungi, Komarov Botanical Institute of the Russian Academy of Sciences, St. Petersburg, Russia; 3 Department of Biological Sciences and Research Center for the Natural and Applied Sciences, University of Santo Tomas, Manila, Philippines

**Keywords:** Particle analysis, *Physarum albescens*, Size distribution, Spore diameter, Computer vision, Spore shape

## Abstract

Measuring spore size is a standard method for the description of fungal taxa, but in manual microscopic analyses the number of spores that can be measured and information on their morphological traits are typically limited. To overcome this weakness we present a method to analyze the size and shape of large numbers of spherical bodies, such as spores or pollen, by using inexpensive equipment. A spore suspension mounted on a slide is treated with a low-cost, high-vibration device to distribute spores uniformly in a single layer without overlap. Subsequently, 10,000 to 50,000 objects per slide are measured by automated image analysis. The workflow involves (1) slide preparation, (2) automated image acquisition by light microscopy, (3) filtering to separate high-density clusters, (4) image segmentation by applying a machine learning software, Waikato Environment for Knowledge Analysis (WEKA), and (5) statistical evaluation of the results. The technique produced consistent results and compared favorably with manual measurements in terms of precision. Moreover, measuring spore size distribution yields information not obtained by manual microscopic analyses, as shown for the myxomycete *Physarum albescens*. The exact size distribution of spores revealed irregularities in spore formation resulting from the influence of environmental conditions on spore maturation. A comparison of the spore size distribution within and between sporocarp colonies showed large environmental and likely genetic variation. In addition, the comparison identified specimens with spores roughly twice the normal size. The successful implementation of the presented method for analyzing myxomycete spores also suggests potential for other applications.

## Introduction

Spores and pollen have evolved to overcome the challenge of dispersal in terrestrial environments, because only very small particles can float in the air. Such propagules are often formed in huge amounts (Li, 2011), may be actively released into the air (Ingold, 1965), assume diverse shapes (Woo et al., 2018), and occur in many unrelated groups of terrestrial organisms with limited mobility, both prokaryotes and eukaryotes (Huang & Hull, 2017). Spores are usually formed on or within fructifications, including in the prokaryotic Myxobacteria (Reichenbach, 1993) and most eukaryotes, such as myxomycetes and myxomycete-like organisms (MMLO, a group comprising various protists, but mainly Amoebozoa (Schnittler, Unterseher & Tesmer, 2006), many fungi (Wijayawardene et al., 2020), mosses, lycophytes, ferns, and seed plants forming pollen grains. The pollen of seed plants shows similar dispersal behavior as spores, but cannot germinate independently, only at the pistil of a receptor plant (Punt et al., 2007). The average size of these propagules is an important trait for species differentiation, even in taxa rich in morphological characters (Brown, 1960). However, only 20–50 propagules are measured in most taxonomic monographs and species descriptions, although a much greater sample size would be desirable for sound statistical analyses.

Spore size is conventionally determined by light microscopy. This is time-consuming and error-prone, depending crucially on the accurate use of the measuring device. Many microscope manufacturers now offer optional camera and software solutions that may be purchased at an estimated minimum price of US$ 4,000. Some of these applications use machine learning algorithms that can be trained to recognize objects and measure them automatically. ZEN (blue edition) by Zeiss is an example. Furthermore, algorithms for range thresholding, simple filters, and edge detection based on intensity change are now widely used. However, these algorithms typically fall short of achieving a segmentation of objects with a complex shape or touching each other. Additional problems are that only a small fraction of the spores on a slide is in focus and that spore densities are too low to measure large numbers. Packing spores more densely leads to high overlap and thus poses problems for the software to separate individual spores optically.

A number of devices have been developed to overcome such limitations. These include the Beckman Coulter particle counter (Brea, CA, USA), which is based on resistive pulse sensing (approximately US$ 38,000); systems based on laser diffraction spectroscopy developed by A. Paar GmbH (Graz, Austria); a system marketed by HORIBA (Kyoto, Japan) based on dynamic light scattering (approximately US$ 45,000); and imaging flow cytometry (IFC) like the FlowCam (Buskey & Hyatt, 2006) as an automated flow-through microscopic device (approximately US$ 65,000). All of these devices except for the FlowCam need to be adjusted for a given application, since the visual inspection of single particles is not possible. A major downside of all these instruments is that acquisition costs are high.

Affordable alternatives are the PlanktonScope (Pollina et al., 2020) and an IFC system described by Göröcs et al. (2018). However, limitations associated with the lower costs typically include the use of a rolling shutter system, which needs an extra algorithm to compensate for the image tearing effect. Moreover, for deep flow cells in particular, the use of entocentric lenses introduces measuring errors, distortion, astigmatism, as well as chromatic and spherical aberrations.

Alternative image analysis tools like scripts, plug-ins, or stand-alone applications based on brightfield microscopy of preserved samples have also been designed (Korsnes et al., 2016; Wagner & Macher, 2012; Vidal-Diez de Ulzurrun et al., 2019; Benyon et al., 1999) to identify and count small particles as well as to determine their morphological features. These approaches enable reliable analyses when contrast between object and background within the field of depth is consistently high and objects are non-overlapping. However, they become unreliable when these conditions are not met.

Here we present a procedure that allows for the reliable low-cost image analysis of spores, pollen, or biological aerosols at high densities. Our procedure is independent of special equipment and proprietary software. Spores are evenly spread on a slide to measure up to 50,000 objects based on photographs taken with a conventional compound microscope. The images are subsequently processed with the open-source software Fiji ImageJ (version 1.52p; Schindelin et al., 2012), which facilitates incorporating plug-ins and machine learning scripts. The workflow allows the visual inspection of every particle. This is often important to ascertain the quality of measurements, since spores are usually mixed with other particles, like pieces of cell wall (anthers, fern sporangia), hyphal fragments (fungi), elaters (liverworts), or capillitia (myxomycetes). We illustrate possible applications on the example of the myxomycete *Physarum albescens* Ellis ex T. Macbr., which releases large numbers of airborne meiospores for long-distance dispersal (Kamono et al., 2009). Specifically, we assessed variation in shape and spore size distribution within a colony and depending on environmental conditions during sporocarp formation. Our goal was to develop a workflow that (1) is based on standard slide preparations routinely used for identification, (2) is independent of special equipment and proprietary software, and (3) measures spore quantities large enough to construct a size distribution graph for robust statistical evaluations.

## Materials and Methods

*Physarum albescens* is a typical member of the dark-spored clade of the Mycetozoa (Leontyev et al., 2019), the slime molds. This group belongs to the Amoebozoa and comprises more than 1,000 described species (Lado, 2005–2020). *Ph. albescens* was sampled throughout the Northern Hemisphere to cover a maximum of intraspecific variation (Rocky Mountains, German and French Alps, Khibine Mountains of the Kola Peninsula, Northern Caucasus, Spanish Sierra Nevada, Kamchatka, see Supplement 1A and Supplement 1B). The life cycle of *Ph. albescens* involves the formation of a colony of usually stalked sporocarps (Schnittler et al., 2012; Stephenson & Schnittler, 2017), each containing between 0.5 to 2.5 million spores (Schnittler & Tesmer, 2008). For each colony, spores of five sporocarps were analyzed.

### Slide preparation

**Figure 1:**
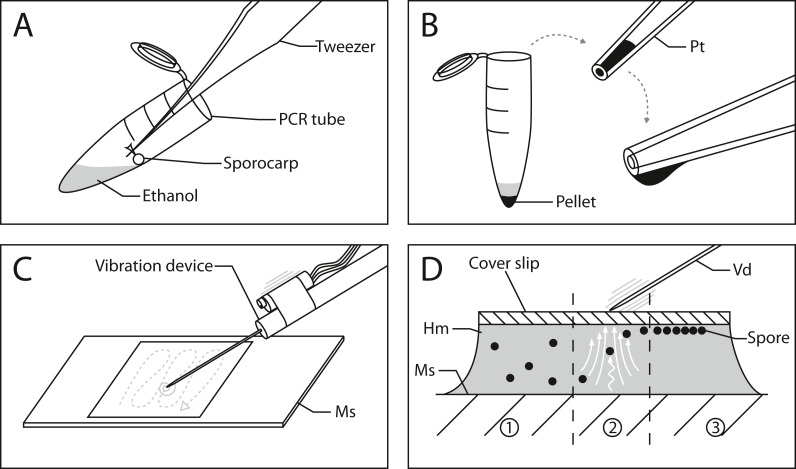
Workflow for spore preparations from *Physarum albescens* sporocarps for quantitative measurements. (A) Step 1: spores are suspended in 20 µl ethanol and centrifuged. (B) Step 2: The pellet containing the spores is transferred to a slide using a pipette tip; the formation of a hanging drop facilitates the evaporation of ethanol to further concentrate the spore suspension before placing it on the slide. (C) Step 3: The spores are arranged in a single layer with an oscillating preparation needle connected to a vibration device. (D) Alignment of spores under the influence of granular convection induced by the vibration device: 1 - untreated spores, 2 - spores under granular convection (white arrows), 3 - spores arranged in a monolayer beneath the coverslip. Abbreviations: Hm, Hoyer’s medium; Ms, microscope slide; Pellet, pellet containing spores; Pt, pipette tip; Vd, vibration device.

Figure 1 outlines the process of slide preparation. One mounted slide was prepared with Hoyer’s medium for each sporocarp (Neubert, Nowotny & Baumann, 1993). The sporocarp was crushed and its spores suspended in 0.2 ml PCR tubes with 20 µl of 70% (v/v) ethanol. Since the spores of myxomycetes (and many other organisms) have hydrophobic ornamentations, ethanol was chosen to avoid spore aggregation. The spores were separated by repeatedly and carefully dipping the sporotheca (stalked fruit body) into the ethanol while holding the stalk with tweezers (Fig. 1A). Spore release was controlled by tilting the tube and dipping the sporotheca into the ethanol at the edge of the liquid. This reduced the risk of submerging and losing significant parts of the capillitium and peridium.

After brief centrifugation (1 min), the supernatant in the PCR tube was removed with a 20 µl pipette tip by capillary forces until the ethanol barely covered the pellet, which was subsequently resuspended. The remaining volume of this concentrated spore suspension must be sufficiently large to be able to transfer it to a microscope slide with a pipette. Slow ejection from the pipette produces a hanging drop so that ethanol can evaporate, resulting in further concentration of the suspension (Fig. 1B). This step is crucial since the ethanol needs to evaporate to the point where no excess liquid is visible when transferring the spores to the slide next to a drop of Hoyer’s medium. The spore mass should be moist, similar in consistence to mud. A spore droplet containing too much liquid will cause the suspension to spill out, rather than being mounted into the medium on the slide. Finally, a coverslip was carefully placed on the deposited droplet, which resulted in a dense spore cluster when the spore suspension and Hoyer’s medium mix.

Vibration was used to disperse the spores in a monolayer. We used a device consisting of a preparation needle attached to a resonance speaker (impedance and power output: 4 Ω by 25 W, no specific manufacturer) connected to a 25 W amplifier (LEPY LP-VS3, Supplement 2). The shaft of the preparation needle is fixed to the shock rod of the resonance speaker by hand or small 3D-printed clamp (Supplement 3). The amplifier is connected to a laptop over the headphone jack or auxiliary port. A frequency signal generator application on the laptop, accessed *via* an internet browser (*e.g.*, https://www.wavtones.com/functiongenerator.php), can be used to generate and control the vibration frequency of the resonance speaker. We initially used 400–600 Hz (sinus wave type) for a pre-alignment of our spores and increased the frequency to 800–1,000 Hz for fine adjustment. Ideally, this sorts the spores into a single layer, thus arranging them at the same height in the z-plane. The pre-alignment and fine adjustment steps each took 1–2 min. The volume regulation on the amplifier and laptop can be used to increase the force of the vibrations. In our configuration, we used 60–80% of the amplifier’s volume and 100% on the laptop.

By applying vibrations generated by this device on the coverslip *via* the needle (Fig. 1C and Supplement 3), a granular convection is induced within the medium. As a result, the spores start to move upwards and align beneath the coverslip (Fig. 1D). Two fingers should hold the coverslip in place during the vibration treatment. Since the vibration only affects a small area around the needle, the vibrating needle is moved over the entire area of the coverslip in a tapping motion to align as many spores as possible (Fig. 1C, gray dashed line). This determines the number of spores that can later be analyzed. The entire process can be monitored under a dissecting microscope. In addition to frequency, vibrations can be adjusted by the pitch angle, roll angle of the device, and the pressure applied to the coverslip.

### Image acquisition and ImageJ pipeline

Microscopic images (resolution 2,880 × 2,048 pixels) were acquired with a Nikon Eclipse 90i compound microscope in the brightfield mode under twentyfold magnification (0.12 µm/pixel on the object plane). The camera system was a Nikon DS-Fi3 with a 1/1.8“ CMOS-color sensor and a pixel size of 2.4 µm. The pixel intensity of the background was set to 90% of the maximum for all three color channels to provide consistent images for the automated segmentation process. For the same reason, the deconvolution function was switched off. The “large-images”-mode of the microscope software (NIS-Elements AR, Version 5.02.03) was used to automatically acquire 144 (12 × 12) single unstitched images. The overlap was set to the lowest possible value to prevent repeated measurements of the same spores at the edges during the subsequent image analysis. The software was set to automatically refocus after every fourth image.

We used ImageJ (version 1.52p) to automate most image processing and analysis steps. ImageJ offers options to use various computer languages. We used mainly Python-based commands on a desktop system with 12 GB RAM and a 3.6 GHz Intel Core i7-7700 processor without GPU processing.

Several subsequent scripts were written to organize, process and later analyze the raw images (Table 1, https://github.com/JanWoyzi/Sporesize-Measurement). Spores were counted and sized in ImageJ using the built-in function “Analyze Particles” (Paana.py, Table 1) or the plug-in “Ellipse Split” (Elli.py, Table 1, version 0.4.0, Wagner & Eglinger, 2017). Both functions work by counting and measuring pixels on a binary image, characterizing clusters of pixels to provide detailed information about spore shape and size. In addition, Ellipse Split approximates for each pixel cluster the ellipse that fits best, which was then used to characterize the objects.

**Table 1:** Scripts used for image analysis. Underlining indicates scripts crucial for the analysis. The column D/S indicates whether calculations are performed on a desktop computer (D) or server (S).

Script name	D/S	Script function
IScore.py v1.0	D	Assesses background intensities (calculates a score for background intensity by mixing RGB values into a grayscale; figure must be comparable for all images)
BScore.py v2.0	D	Calculates a score for spore density from the brightness of the total area covered by spores
CreatingDir.py v2.0	S/D	A script designed to build directories for scripts (for instance, Elli.py) that cannot create them automatically
Structurefilter.py v3.0	S/D	Detects edges of and sharp angles between objects to separate them by a regional watershed line; the result is an image with overlaid separation lines between spores; necessary for handling images with high spore density
Weka.py v3.2.33	S/D	Machine Learning algorithm, works with a pre-trained model to recognize objects (as differently colored patches of pixels); the result is a classification probability of each pixel as spore or background (more than two classes can be defined as well); this is presented as a stacked image (one for each class)
Destacker.py v4.0	S/D	Separates the WEKA segmentation stack (here two classes, i.e., spores and background) and saves the respective results
Paana.py v6.0	S/D	Particle Analyzer, analyzes spore shapes, describes features like circularity, roundness, etc. (does not use a pre-defined shape, therefore more sensitive but requires precise segmentation)
Elli.py v2.0	D	Ellipse Split plugin (alternative algorithm to Paana.py), analyses the spore shapes according to a pre-defined ellipsoid shape (less sensitive to a particular shape, since measurements are based on approximated ellipses), more robust in case of segmentation errors
RGBROI.py v2.0	S/D	Extracts area-related object features (e.g., RGB values) by comparison with the raw image

We employed a plug-in called “Trainable WEKA segmentation” (version 3.2.33, Arganda-Carreras et al., 2017) based on Waikato Environment for Knowledge Analysis (WEKA) toolsets with various machine-learning algorithms to perform functions like clustering, classification, regression, visualization, and segmentation. However, other open software for segmentation and classification (*e.g.*, Ilastik, described in Berg et al., 2019) can be used as alternatives to WEKA. We chose WEKA because it could be easily implemented in our script workflow with ImageJ without using too many different software applications.

To train the tool, the user takes an example image and visually marks about 10 target objects (spores) and non-target objects (background) on an exemplary sample image. Additional classes of objects (*e.g.*, branches of the capillitium or remains of hyphae) can be defined to delimit contaminations, but this approach will increase error rates when separating the target objects from the background. For most applications, it will be more effective to exclude these objects at a later stage of the analysis. The best segmentation results in terms of processing time per image were achieved with small and straightforward models and fine-tuned filter sets. Not only can the number of segmentation classes and filter sets be adjusted, but the complexity of the decision tree can also be freely modified. Our model has only two segmentation classes and uses a minimum of filter sets (see Github repository). After a few iteration steps, after which the user can correct the decisions made by the algorithm, the result can be saved as a model to be applied to additional images in a batch process.

**Figure 2:**
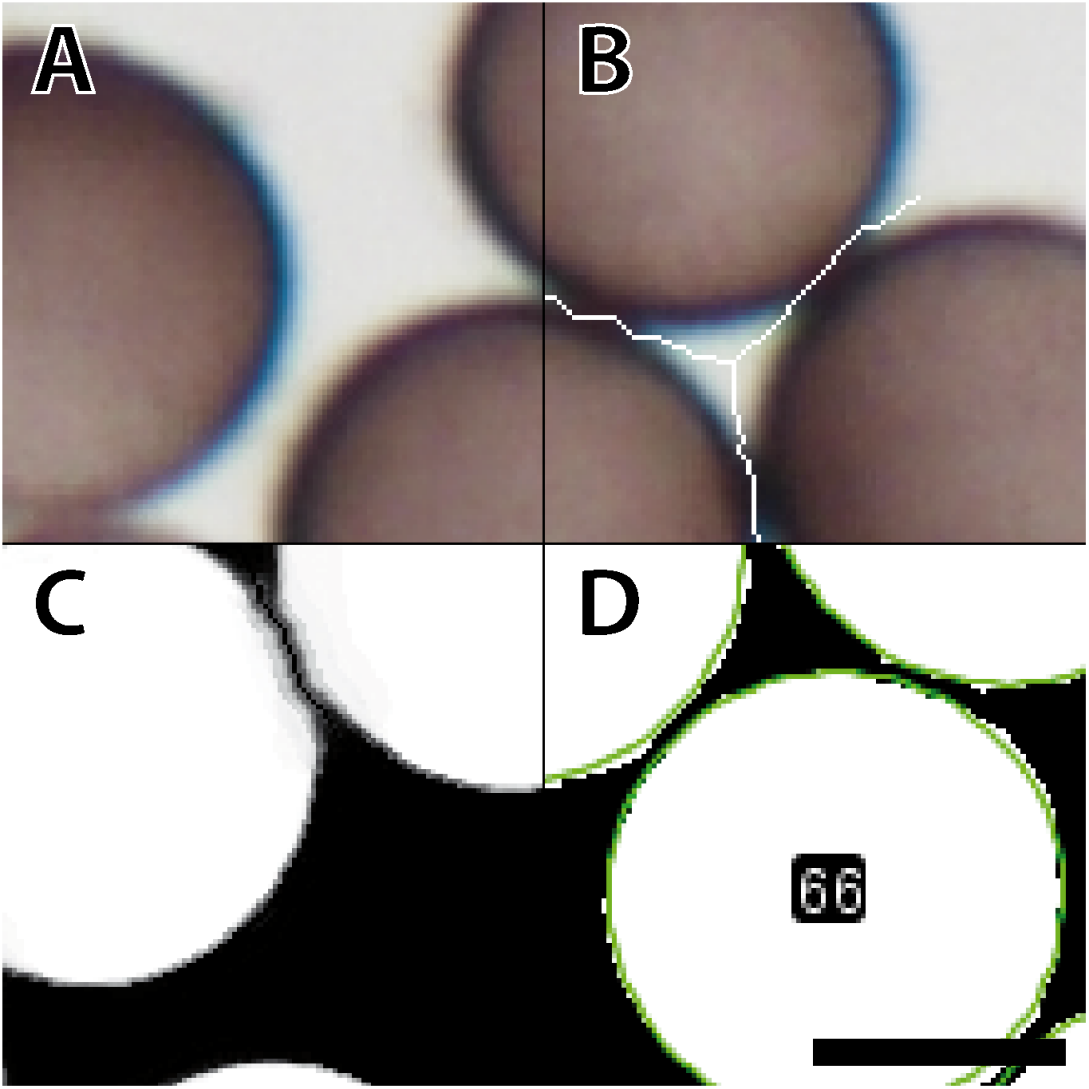
Workflow for image processing of *Physarum albescens* spores. (A) Raw image, (B) structure filter applied, (C) segmented image, and (D) spore shapes selected with the procedure “Ellipse Split”; at this stage an ID is given to every single spore (example: no. 66). These operations are carried out simultaneously on all spores of a given image. Bar = 6 µm.

Figure 2 shows the entire segmentation process for a few spores. A consequence of high spore densities on the images (Fig. 2A shows a small section) is that segmentation with the WEKA plug-in generated spore clusters. These clusters could not be resolved by standard watershed methods (Soille & Vincent, 1990), which would falsely identify two adjacent spores as a single object. To prevent such false assignments, we created an overlay of each image where lines one-pixel wide and in background color separate the individual spores. The separating lines were computed by combining the plug-ins “FeatureJ” (version 3.0.0, Meijering, 2015) and “BioVoxxel” (version 12. Brocher, 2012). “FeatureJ structure” calculates for each pixel the eigenvalue of its structure tensor. For two spores that touch each other, the pixels in the two areas in the acute-angled corners have the highest eigenvalues. The function “Watershed Irregular Features” was used to connect only the center points of areas that are close enough to belong to adjacent spores, using a pre-defined maximum radius as a criterion. The resulting connected lines (Fig. 2B) were overlaid on the raw images, separating spores in high-density areas, and were successfully segmented by the WEKA plug-in (Fig. 2C). Figure 2D shows the result of the subsequent analysis with the Ellipse Split tool. The fitted shapes for each spore (green circles in Fig. 2D) are then subject to further analysis. The script can run in batch mode to process a series of images for just one or many specimens.

Initial tests and the segmentation problems encountered with images characterized by high spore densities revealed the need for a robust approach to estimate spore densities on the images. This approach was then used to exclude critical images where high spore densities would lead to erroneous measurements or, conversely, very low densities would lead to errors in some analysis functions in ImageJ. We established the proxy “BScore” to measure the overall brightness of the entire image. Due to the dark spores and white background under brightfield conditions, high spore densities lower the overall brightness. By delimiting the two extreme conditions, no spores *versus* spores filling (nearly) the entire field, the approximate boundaries can be set and used as filters both during the segmentation step and later during the statistical analysis.

**Figure 3:**
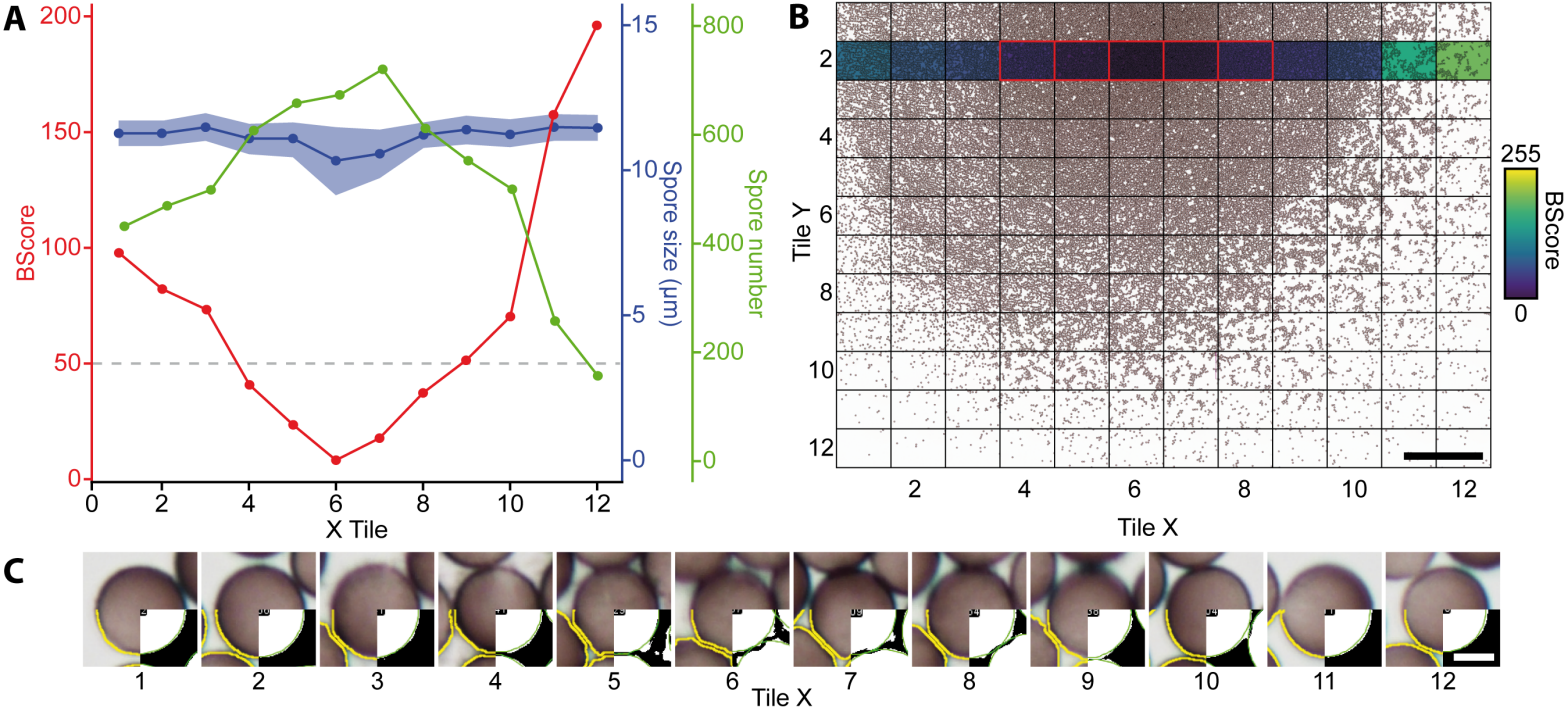
Analysis of a mounted slide shown are the parameters for a belt of 12 images (43,540 spores in total) taken from a slide with varying spore densities. (A) BScores (red line), spore numbers per image (green line), spore diameter (blue line with shaded standard deviation), and threshold for excluding an image based on the BScore (gray dotted line). (B) Overview of a slide showing spore densities on 144 stitched images (bar = 500 µm); the second row with the colored background was selected to generate the graph shown in A. Tiles framed in red have a low BScore and were thus excluded. (C) Examples for watershed-separated spores from 12 images (bar = 6 µm) differing in spore densities; the upper half of each image shows the raw image, the lower left quarter the final selection obtained with the particle analyzer (yellow line), and the lower right corner the final segmentation obtained with the Ellipse split function (green line).

**Figure 4:**
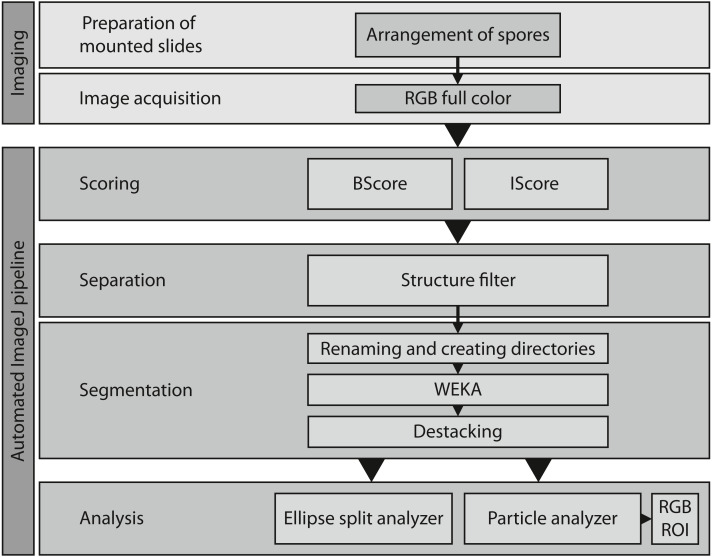
Overview of the entire workflow from sample preparation to data acquisition. See Table 1 for the respective scripts.

Figure 3 shows a slide with a high spore density in the center, illustrating subsequent segmentation problems. To eliminate such cases, two scripts for image analysis were created (Github repository). The first, called IScore, uses the background intensity of an image as a proxy to ensure consistent image acquisition. The second, named BScore, generates a proxy value for spore density based on which images with extremely high spore densities are identified and discarded (Fig. 3). Critically low BScores result from massive spore clusters or when spaces between densely packed spores are filled by fine amorphous particles. These situations can be avoided by reapplying the vibration device to spread the spores more evenly and thus limit the number of images to be excluded based on a low BScore (0–2 per slide). Figure 4 and Table 1 show the entire workflow and the respective scripts for the image analysis. The final result is an array of shape features for each object such as area, circularity, aspect ratio and maximum diameter (Table 2). In addition to determining spore size and shape based on this information, specific regions of interest can be selected for every single spore and be used with the raw images to measure RGB values as a proxy for spore coloration.

**Table 2:** Overview of all measured spore features included in the data set.

Column name	Description
Indiv	Sample name
Label	Complete identification name
Area	Area in pixel
Perim.	Perimeter in pixel
Major	Major axis in pixel
Minor	Minor axis in pixel
Circ.	Circularity = 4π*area/perimeter^2^
AR	Aspect ratio between major and minor axis
Round	Roundness = 4*area/(π*major_axis^2^)
Solidity	Area of a particle divided by its convex hull area
Mean_r	Mean intensity value^*^
StdDev_r	Standard deviation of the intensity value^*^
Mode_r	Mode intensity value^*^
Min_r	Minimum intensity value^*^
Max_r	Maximum intensity value^*^
X	Coordinate of the image on the *x*-axis
Y	Coordinate of the image on the *y*-axis
Index	Image number within a sample
BScore	Brightness score
Type	Object classification based on size

**Notes.**

* Recorded for all red, green and blue channels, but shown only for the red channel (r) to avoid repetition.

### Calibration

A range of parameters can be adjusted (*e.g.*, overall spore density, contrast, and homogeneity of background intensities) to distinguish spores from the background and from one another. The arrays summarizing the features of each captured object must be filtered to exclude non-target objects (capillitium, hyphal fragments, air bubbles and amorphous particles; Supplement 4). This can be checked at any stage, since each object can be retrieved with ImageJ by using its unique label or address given by the scripts.

These settings are species-specific and hence need to be adjusted for every new application.

This critically requires measuring many spores with sufficient precision instead of striving for the highest possible precision for just a few spores. Consequently, we used only 20× objectives (NA: 0.5, Nikon Plan Fluor), resulting in a resolution of 0.12 µm/pixel. However, higher magnification may have to be used for smaller objects. The most critical step in the process is the slide preparation since spores have to be densely distributed to allow many spores to be photographed at once, but the vibration treatment should aim to arrange all spores in a single plane.

The image acquisition and analysis can be automated, using the same settings for single species or a group of related species. For *Ph. albescens*, we used a light intensity of 225 of 255 scale units, resulting in an exposure time of 15–16 ms, 20 × magnification, variation of the IScore between images ≤10%, and a BScore ≥50. The removal of non-target particles can be optimized in an iterative process, based on the compiled array of object features. Useful criteria are particularly the minimum area to exclude small amorphous particles), the aspect ratio to exclude remnants of the capillitium and air bubbles, and circularity or roundness.

For *Ph. albescens*, we expected spore sizes between 8 and 15 µm. Therefore, we removed all particles exceeding 1,500 pixels (equivalent to a sphere diameter of 5.25 µm), or showing an aspect ratio >2 or a circularity <0.7. These settings are likely to be species-specific.

### Data analysis

The results of the image analysis (Table 1 and Fig. 4) are saved as csv-files for subsequent analysis with statistical software such as R (R Core Team, 2017). Between 10,000 and 50,000 spores per slide (Supplement 1) were measured and a frequency density plot was constructed. To compare specimens within a region, we calculated the mean absolute deviation (MAD) between specimens as:

$MAD= \frac{1}{n} {\mathop{\sum }\nolimits }_{i=1}^{n} \left\vert {x}_{i}-\bar {x} \right\vert $, where *n* is the number of data points, $\bar {x}$ the mean of the individual values *x*_*i*_. To analyze the resulting distribution plots, we calculated a function for each sample with the built-in density function of R to automatically locate all local maxima and minima in the data set. Setting frequencies thresholds allowed us to exclude rare occurrences of non-target objects and classify different types of objects (*e.g.*, amorphous particles, normal-sized spores, oversized spores, large amorphous particles). These object classes can also be fit to different distribution families, in our case the gamma distribution (Supplement 1C).

To compare the performance of our automated approach with that of standard manual measurements, we measured the diameter of 100 randomly selected spores from an image with 520 spores of *Ph. albescens*, specimen Sc29313. To test for the effect of sample size, we compared results obtained for 25, 50, or all 100 spores. A second treatment group with three levels was used to test for precision. These were (1) manual measurement at the standard magnification of 20x, (2) manual measurement at an additional 8x digital zoom, and (3) application of the automated measuring algorithm. For manual measurements, the manually selected vertical distance between spore margins (without ornaments) was measured as spore diameter. We repeated all measurements five times. Additionally, automated measurements of all 520 manually measured spores were compared with those of all 144 images acquired for this specimen (35,176 spores in total). However, the latter two data sets were not included in the statistical test. A linear mixed model was used to test for the main and interactive effects of digital magnification (zoom level), numbers of analyzed spores and repeated measurements (Table 3). A pairwise least-square means *post hoc* test (R package: Emmeans, version 1.6.2-1) with false discovery rate correction (Benjamini & Hochberg, 1995) was applied to identify significant main and interaction effects between pairs. The repeated measurements of spores were treated in the model as a random variable.

**Table 3:** Results of Type III Analysis of Variance with degrees of freedom calculated according to Satterthwaite’s method. Effects of digital magnification (Zoom), spore numbers analyzed, and number of replicate counts and their interactions on spore diameters.

Source of variation	Sum of squares	Mean square	NumDF	DenDF	F	*P*-value
Zoom	6.10	3.05	2	2481.0	111.22	<0.001
Spore number	0.71	0.35	2	2494.2	12.92	<0.001
Replicate	0.11	0.03	4	2481.0	0.99	0.413
Zoom:Spore number	2.23	0.56	4	2481.0	20.38	<0.001
Zoom:Replicate	0.57	0.07	8	2481.0	2.59	0.008
Spore number:Replicate	0.82	0.10	8	2481.0	3.72	<0.001
Zoom:Spore number:Replicate	1.59	0.10	16	2481.0	3.67	<0.001

Furthermore, we analyzed the spatial distribution of different spore sizes with 25,000 Monte–Carlo simulations of spore position within the 12 by 12 image matrix of sample Sc24902, and calculated Moran’s I to assess the spatial displacement effectiveness of the vibration device on different spore sizes.

## Results

### Spore size distribution

**Figure 5:**
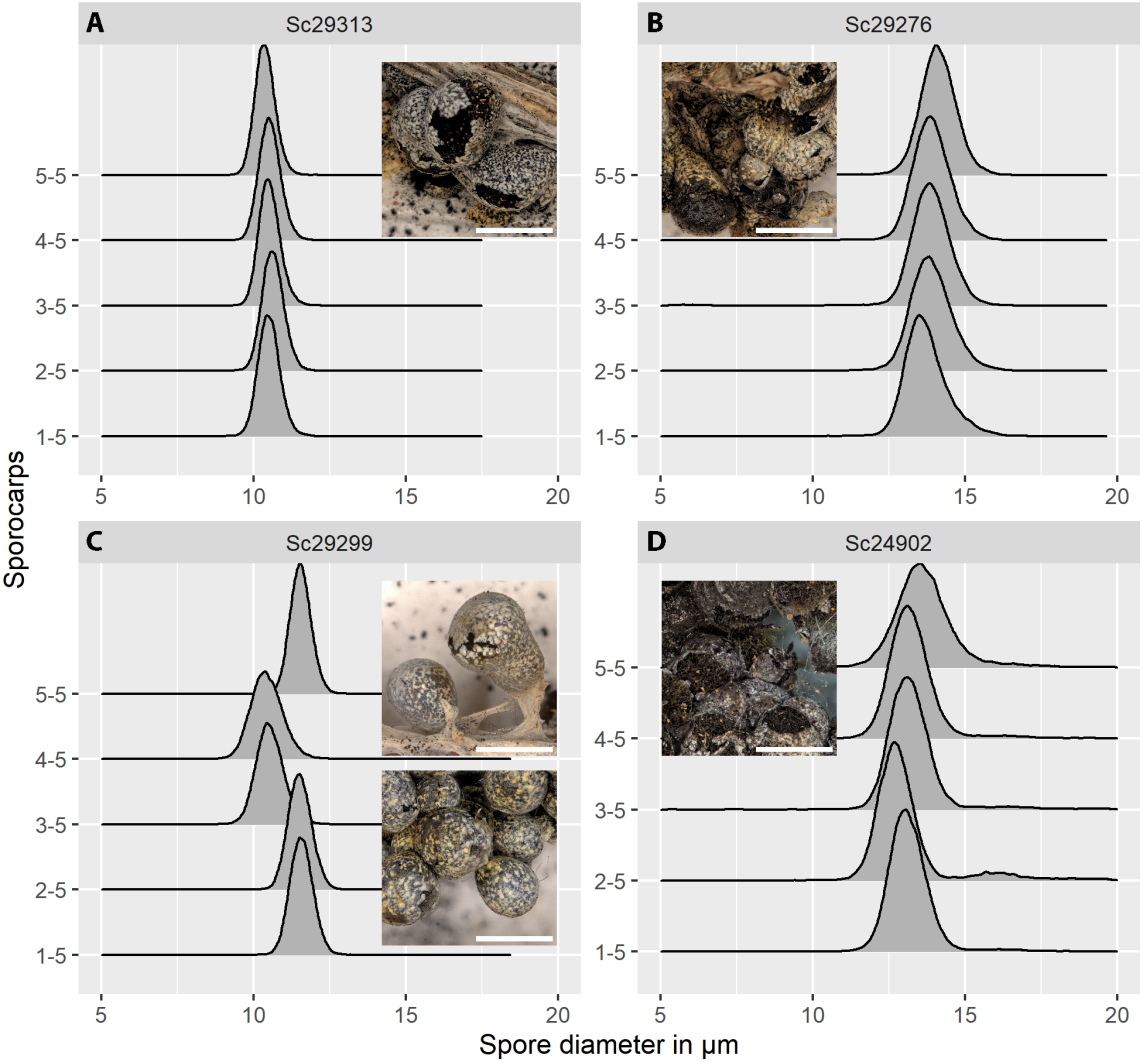
Spore size distributions (black line: density function) for five sporocarps from each of four strains of *Physarum albescens*. (A) Sample Sc29313, well-matured sporocarps. (B) Sample Sc29276, sporocarps nearly mature. (C) Sample Sc29299, a mixed colony with sporocarps belonging to two genetically different individuals (sporocarps 1, 2, and 5 *vs.* 3 and 4). (D) Sample Sc24902, including a noticeable fraction of oversized spores. Images show the appearance of the respective specimens, note the densely crowded sporocarps with poor calcification in sample Sc24902. Bar = 1 mm (magnification = 20x).

The large number of counted objects made it possible to define extremely narrow size classes, resulting in quasi-continuous distributions for spore size (Fig. 5). The spore size distributions can be approximated by a Gamma function (Figs. 5A–5D, Supplement 1A, Supplement 1C). Altogether we processed 7.5 million spores, on average 31,000 per sporocarp. Diameters ranged between 10.1 (Sc29307, Rocky Mts.) and 13.6 µm (Sc29276, Rocky Mts.) with an overall mean of 11.9 µm. The average standard deviation within a sample (sporocarp level) was 0.48 µm (range 0.33–0.90 µm). The skewness of the approximated Gamma function is a measure of overall spore size uniformity. High positive values indicate the frequent occurrence of oversized spores, as evident in specimen Sc24902 (Caucasus Mts.) with a skewness >0.5 and a bimodal spore size distribution (Type 1: normal-sized spores, Type 2: oversized spores). Colonies from the Kamchatka Peninsula and the Caucasus showed highly uniform and narrow distributions (mean absolute deviation between specimens of 0.35 and 0.32 µm, respectively; Supplement 1D). Colonies from the Rocky Mountains were less uniform (mean absolute deviation of 0.99 µm) and included both the smallest and largest spores of all analyzed strains.

The distribution of spore sizes was asymmetric in many specimens, especially in spores that appeared not to be fully mature (Figs. 5B, 5D). Therefore, the best measure of average spore size was the peak of the fitted Gamma-distribution, rather than the mean diameter (Supplement 1C), which coincides with, or is close to, the modal value, *i.e.,* the size class containing the largest number of objects. Figure 5C shows a case of two co-occurring individuals sampled in a mixed colony of sporocarps, where the spore size distribution of two sporocarps differs from that of the other three.

**Figure 6:**
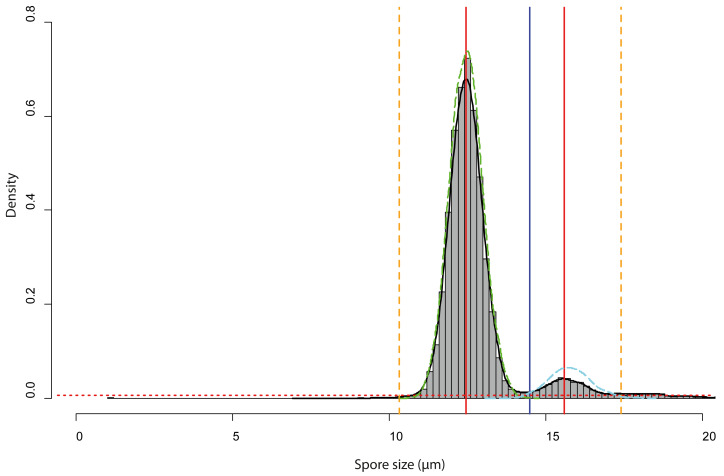
Analysis of the bimodal spore size distribution for Sporocarps 2–5 of *Physarum albescens* specimen Sc24902. The histogram in grey (bin width ca. 0.10 µm) and the density function (black line) displays the spore size distribution. The red dotted horizontal line depicts the threshold at 0.000004 frequency density units and works as a high-pass filter. The red vertical line is the position of the highest frequency. The vertical blue line shows the minimal local frequency between the two peaks. The orange dashed vertical lines indicate the left and right limits of the analyzed interval, which were set as intersects between the thresholds for particle detection and the density function. The green and cyan dashed lines are the density functions of the fitted spore size distribution of the normal-sized and oversized spores, respectively.

Spore size distributions of some sporocarps, for example, sporocarp 2–5 shown in Fig. 5D could be fitted to two overlapping gamma functions (Fig. 6) with two peaks, one for normal-sized and one for oversized spores. The total spore volumes corresponding to the two peaks (1,013 and 1,959 µm^3^) were equivalent to a ratio of 1:1.93. A Monte Carlo simulation of Moran’s I (25,000 simulations) showed the I-value to be significant (0.69, *p* <  0.001) for normal-sized spores (12.9 ± 0.6 µm diameter) in the total scanned area (12 × 12 images covering 12.2 mm^2^), indicating clustering. For oversized spores (15.9 ± 0.5 µm diameter), the I-value was not significant (−0.10, *p* = 0.99), indicating a random distribution of spores for this size class.

### Accuracy of the method

**Figure 7:**
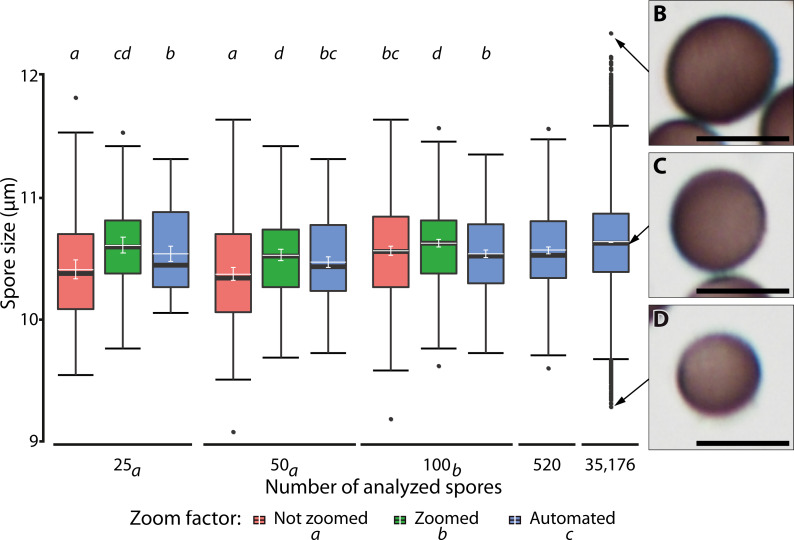
Ranges of spore size for manual and automated measurements. (A) Box plots of spore size in *Physarum albescens* determined by manual and automated measurements (box: 25th to 75th empirical quartiles, black whiskers: 1.5 times the interquartile range, black thick horizontal line: median, dots: single outliers, white horizontal line: mean, white whiskers: 95% CI). Varying numbers of spores were measured on single images (all up to 520 spores) and 144 images (last box, 35,176 spores). Different lower-case letters in the legend indicate significant differences in spore size in pairwise *post hoc* tests between zoom levels. Lower case letters on the upper facet level represent a grouping results of pairwise *post hoc* test according to the numbers of spores measured. Letters on the lower facet level refer to post hoc grouping results for zoom levels grouped by amount levels. (B–D) Examples of individual spores representing (B) upper outliers (oversized spores), (C) average-sized, and (D) lower outliers (unusually small spores), with the arrows pointing to the respective diameter in (A). Bars = 10 µm.

Figure 7 shows the results of different manual and automatic measurements of spore size from the same sample. For automated measurements including all images, 95% confidence interval of the means were very small (10.62–10.63 µm, in Fig. 7, last bar), which was consistent with the spore size measurements of various samples (Supplement 1B). This low variation is a direct effect of the large number of objects measured, which also increases the number of extreme outliers (Fig. 7, compare results from 520 spores with those from a total of 35,176 spores sized on 144 images).

A linear mixed model (Table 3) revealed a significant three-way interaction among zoom level, the number of analyzed spores, and repeated measurements (F(16, 2,481) = 3.63, *p* < 0.0001) for spore diameter, indicating that all factors influenced each other, including effects of human error in delineating spore margins. All two-factor interactions were also statistically significant, indicating that the factors in all pairwise combinations influenced each other as well. Differences among zoom levels (F(2, 2,481) = 111.2, *p* < 0.0001) and number of spores analyzed (F(2, 2,494.2) = 12.9, *p* < 0.0001) were highly significant, but not among repeated measurements (F(4, 2,481) = 0.99, *p* = 0.41). The *post hoc* analysis showed that the measured spore diameter fell into two groups, depending on the number of measured spores. Results for 25 and 50 measured spores were similar and resulted in significantly (*p* < 0.001) smaller spore diameters than for 100 measured spores. Differences among zoom levels were as well significant (*p* < 0.0001). Manual measurements without magnifying the spores with a digital zoom resulted in the smallest mean spore diameters, whereas additional digital magnification produced the largest spore diameters. Automatic measurements resulted in intermediate values. The *post hoc* analysis of significant interaction effects between zoom levels and number of analyzed spores showed that manual measurement without the digital zoom and low spores numbers led to significantly smaller average spore sizes (*p* < 0.001). Regardless of the number of measured spores, all automated measurements grouped with 100 manually measured spores (without zoom) and resulted in intermediate spore sizes (Fig. 7).

## Discussion

Spore size is an important taxonomic character in many organisms. Myxomycetes are no exception. Their spores are mostly spherical, rarely ovoid, and ornamented. According to the descriptions given in standard monographs, size varies little within species (<1 µm in diameter, except for oversized spores) but greatly among species, with sizes ranging from 4–22 µm, although for >80% of all species the range is narrowed to 7–12 µm (Schnittler & Tesmer, 2008). This small variation indicates that spore size is under strong evolutionary constraints. The smaller a particle, the greater its dispersal potential (Norros et al., 2014), since its terminal velocity is inversely related to the power of its radius according to Stoke’s law. The workflow outlined here assigns a shape to each photographed spore, which allows analyzing subtle differences not only in spore diameter, but also in spore characteristics such as area, perimeter, circularity, and coloration (Table 2).

### Accuracy of the method

Manual measurements of small particles such as spores are tedious, even when assisted by an image analysis tool. Proprietary software marketed with expensive high-end microscopes offer suitable working environments to analyze such particles, with many possibilities offered to integrate different machine learning approaches (*e.g.*, ZEN, blue edition of Celldiscoverer 7 from Zeiss, https://www.zeiss.de/mikroskopie/produkte/mikroskopsoftware/zen.html; Apeer, also from Zeiss, https://www.apeer.com/home; or Leica imaging software, https://www.leica-microsystems.com/products/microscope-software). Nevertheless, it remains challenging with manual measurements to correctly determine the major and minor axis of non-spherical spores, like those of most ascomycetes.

Here we present an alternative to manual measurements, aiming primarily to maximize the number of objects measured rather than focusing on high-precision measurements of spore dimensions. The reason for this choice is that variation induced by the biological processes leading to spore formation, such as mitosis or meiosis that influence spore cleavage, is always much greater than measurement errors. Therefore, we used only a 20-fold objective for magnification, although the method would work equally well for higher magnifications. Greater magnification would slightly improve precision but cover significantly fewer spores per image. Thus, the accuracy of our method exceeds that of careful manual measurements and maximizes the number of analyzed spores.

The main qualitative difference of our method compared to manual measurements is that the shape (and color) of each object can be determined separately. This may be achieved by fitting the analyzed objects to a pre-determined shape like an ellipsoid (script Elli.py, Table 1), or by a more flexible procedure (Paana.py) that approximates the real shape of an object. Accuracy of the measurements is critically influenced by the watershed process separating objects that touch each other. This requires spores to be arranged in a monolayer on the slides because overlapping spores cannot be adequately separated. The script BScore.py we used has been designed to ensure this condition is met. Overlapping spores introduce systematic errors by underestimating mean particle size and inflating variation (Fig. 3A, x-tiles 4–8). Consequently, a balance must be found between spore densities low enough to ensure high accuracy (at the expense of effectiveness) and high enough to increase efficiency (but risking high spore overlap).

Spores of different sizes respond slightly differently to the vibration device we used. The resulting sorting effect leads to a better random distribution of the oversized (Moran’s I = −0.1, *p* = 0.99) compared to the smaller spores. Larger spores appear to be more susceptible to the applied vibration and move further than the smaller spores (compare Figs. 7B–7D). The high and highly significant I-value (0.69, *p* < 0.001) for normal-sized spores can be explained by the interaction between the highly concentrated spore suspension and Hoyer’s medium when placing the cover slip on the suspension. It is thus recommended to analyze numerous images (144 in our case) at different positions of the spore cloud (Fig. 3) to avoid biased sampling. However, the sorting effect is certainly much smaller than the range of biological variation, as also suggested by the similar number of outliers (*i.e.,* extremely small or extremely large objects) in all images analyzed in Fig. 3A.

A third challenge is the exclusion of non-target objects, like capillitium parts in myxomycetes or hyphae in true fungi. This is achieved by defining thresholds for object features such as shape, size, or volume. A big advantage of a microscope-based workflow, such as ours, compared to the use of particle counters is that every single object can be manually inspected. Therefore, the respective filtering procedures can be refined on stored images in several iterative rounds of analysis to improved data reproducibility, independent from any human biases.

### Automatic recognition of objects by machine learning

Machine learning algorithms allow fast and reliable object recognition (*i.e.,* separation from a background) in different biological systems such as cell growth in plants (Galotto, Bibeau & Vidali, 2019) or moth species identification (Feng, Bhanu & Heraty, 2016; Feng et al., 2016). The model we trained and integrated into a batch workflow in WEKA is exceptionally fast and can be used with a few single images only. The only strict requirement is that the image acquisition setup (*i.e.,* exposure time, light intensity, etc.) is highly standardized. However, complex tasks like identifying single spores in a tight cluster (touching or overlaying each other) require more elaborate machine learning approaches and cannot be done by WEKA segmentation. As a solution, we developed a vibration-based sample preparation procedure to arrange the objects on a single plane and applied a separation filter by combining the plug-ins “FeatureJ” and “BioVoxxel” in ImageJ (see Materials and Methods). The generated segmented objects revealed a number of distinguishing features such as area, circularity, aspect ratio and maximum diameter (see Table 2) for every object and ensured large sample sizes and extremely small confidence intervals (see above). As a result, the estimated median spore sizes in large samples approached the true mean of the entire spore population (Fig. 7A).

### Possible applications

Spore size is an important criterion in taxonomy. Therefore, exact measurements may help to identify cryptic biological species (*i.e.,* reproductively isolated sympatric populations), for example in myxomycetes, as found in a number of molecular studies (Feng & Schnittler, 2015; Feng, Bhanu & Heraty, 2016; Feng et al., 2016; Shchepin, Novozhilov & Schnittler, 2016; Dagamac et al., 2017; Janik, Lado & Ronikier, 2020). Figure 5C shows such a case, where colonies of two different phylogroups (*i.e.,* putative biological species) growing together differ in spore size distribution. Furthermore, where fructifications develop rapidly, such as in myxomycetes (often within 1–5 days, Schnittler, 2001), weather conditions may strongly influence spore size. For example, snowbank (nivicolous) myxomycetes like *Ph. albescens* (Ronikier & Ronikier, 2009) develop fructifications under the edges of melting snowbanks where temperatures notably increase within hours (Schnittler et al., 2015). Figure 5B shows less regular size distributions of spores as a possible consequences of such unsuitable environmental conditions, which could be due to impacts on spore cleavage.

Quantitative methods like ours are also suitable to estimate the proportion of oversized spores (“macrospores”). Samples with a significant fraction of oversized spores are fairly common in nivicolous myxomycetes and are often described as forms on their own. Poulain, Meyer & Bozonnet (2011) list macrosporous forms within the genera *Lamproderma* (4 of 22 nivicolous species, plus the enigmatic *L. acanthosporum* with extremely large spores) and *Meriderma* (4 of 8 species with macrosporous forms). Such a case is shown in Figs. 5D and 6, where a population of spores with the roughly twofold volume of normal spores can be distinguished. Quantitative investigations are needed to prove if these macrosporous varieties represent indeed separate taxa. Even more interesting, what is the genetic constitution of such spores? Are they multinucleated (as shown for myxomycetes, Novozhilov et al., 2013), or do they represent restitutional diploid nuclei after an aberrant meiosis? There is evidence that in myxomycetes, meiosis usually occurs after spore cleavage inside the young spores (Clark & Haskins, 2013). If the second hypothesis can be proven, would such nuclei give rise to diploid amoebae and initiate an asexual life cycle, resulting in homothallic clones (Collins, 1979; Collins, 1981)? Apparently, asexual (non-heterothallic) strains (see discussion in Feng, Bhanu & Heraty, 2016; Feng et al., 2016; Walker & Stephenson, 2016) were found in many cultivable myxomycetes (Clark & Haskins, 2010).

Our method can also be used to answer an old question: does genome size influence spore size? For flowering plants, ploidy level (and thus DNA content) is often positively correlated with pollen grain size in polyploid evolutionary lineages of related species (*e.g.*, Katsiotis & Forsberg, 1995; Marinho et al., 2013; Möller, 2018). Although we are not aware of similar studies for myxomycetes, chromosome numbers appear to vary as well, at least within larger groups (Hoppe & Kutschera, 2014), suggesting that such a positive relationship could also exist in myxomycetes.

Our first results on myxomycetes demonstrate that quantitative analyses of spore size and shape may help to answer this and many similar biological questions. Large amounts of spores are often easy to obtain, like crushed sporocarps of myxomycetes, spore prints in mushrooms and ferns, spore bearings in plant parts infected by smuts and rusts, or anthers in flowering plants, which facilitates such investigations.

### Limitations of the method

Myxomycetes have nearly spherical spores, unlike many asco- and basidiomycetes. Structures protruding from spores or pollen grains, like bulged germination pores or a hilus where basidiomycete spores were attached to the sterigma, may also cause difficulties. Only for a small fraction of spores, these structures will be situated in the optical plane, and the apparent spore size will hence vary. Fitting objects to a pre-defined structure, like an ellipsoid, may help overcome such problems, because such structures will be ignored when they are situated in the optical plane. A major challenge that remains, however, is to estimate the shape of irregular objects from only one 2D projection.

The analysis of spores with conspicuous ornamentation such as spines (*e.g*., *Meriderma spinulisporum*) or a reticulum of elevated ridges (*e.g.*, *M. cribrarioides*, Poulain, Meyer & Bozonnet, 2011) requires a more sophisticated machine learning algorithm. In our example, the outer limit of a spore is simply defined as the steepest gradient in contrast. To measure the size of spores with ornamentations being ignored, a mathematical correction for the average extension of the ornaments could be applied. A lack of optical contrast may also be a problem. However, very pale objects can be stained or analyzed by using phase or differential interference contrast. Commonly applied dyes, such as Melzer’s reagent for fungi, can solve the problem of poor contrast but may introduce other uncertainties, such as inflated objects (*e.g.*, McKnight, 1968) or altered shapes.

## Conclusions

The quantitative method presented herein allows analyzing a large number of spores that can usually be quantified and sized only by employing expensive equipment. The method is based on the image analysis of monolayered spores in simple slide preparations. It proved highly reliable compared to manual measurements, which enabled us to demonstrate intraspecific variation in the average spore size of different individuals of *Ph. albescens*. A cohort of spores having twice the volume of normal spores could be precisely quantified. Various applications of the method are conceivable, particularly with other spore-bearing organisms.

## Supplemental Information

10.7717/peerj.12471/supp-1 Supplemental Information 1 Accessions of all specimens of *Physarum albescens* used for automated spore analysis Given are collection numbers (collection M. Schnittler) and the respective locality.
S1a **Spore measurements of single sporocarps.** Shown are number of spores measured, mean diameter, standard variation (SD), and skewness of spore diameter distribution for 3–9 sporocarps from 51 strains of the myxomycete *Physarum albescens*. Type 1 spores have normal size, Type 2 spores are oversized.
S1b **Grand means of spore numbers.** Shown are spore numbers for all sporocarps measured, diameter, standard variation (SD), skewness of the spore diameter distribution, and the mean absolute deviation between individual sporocarps for 51 strains of the myxomycete*Physarum albescens.* Type 1 spores have normal size, Type 2 spores are oversized.
S1c **Measurements of selected sporocarps are shown in Fig. 5.** Listed are the accession number, number of the individual sporocarp, number of spores measured, spore classification (1: normal sized, 2: oversized spores), the mean diameter ± ‘SD, and the parameters for the fitted gamma function of the shape.
S1d **Comparison between regions.** Mean absolute deviation of spore size of all specimens of *Physarum albescens* used for automated spore analysis, grouped by regions. Type 2 spores were excluded.

10.7717/peerj.12471/supp-2 Supplemental Information 2 Shopping list to build the vibration device

10.7717/peerj.12471/supp-3 Supplemental Information 3 Fully assembled vibration device and holding suggestion (A) Fully assembled vibration device with amplifier, resonance speaker, 3D-printed clamp and preparation needle, (B) and (C) holding position of the vibration device without the 3D-printed clamp.

10.7717/peerj.12471/supp-4 Supplemental Information 4 Example of an air bubble as a non-target object to be filtered out (A) Original image, bar = 25 µm. (B) Segmented image, recognized target objects in yellow. (C) Target objects labeled. Note the absence of labels for the air bubble and included small amorphous particles.

## Additional Information and Declarations

### Data Availability

The following information was supplied regarding data availability: Further and detailed measurements are available in the Supplemental File. The scripts or data are available at GitHub: https://github.com/JanWoyzi/Sporesize-Measurement.
