# Supplementary figures and images for: A workflow for low-cost automated image analysis of myxomycete spore numbers, size and shape
Source: PeerJ. 2021 Nov 16;9:e12471. doi: 10.7717/peerj.12471 (PMC8605758; doi:10.7717/peerj.12471)

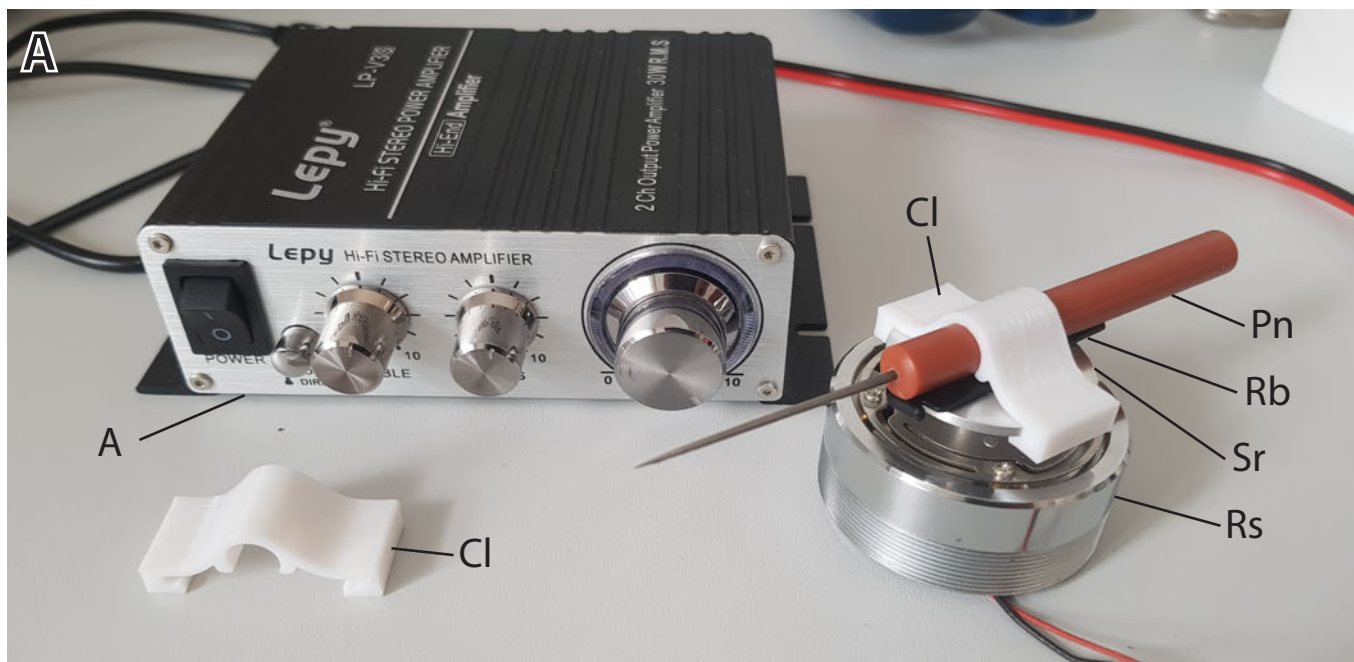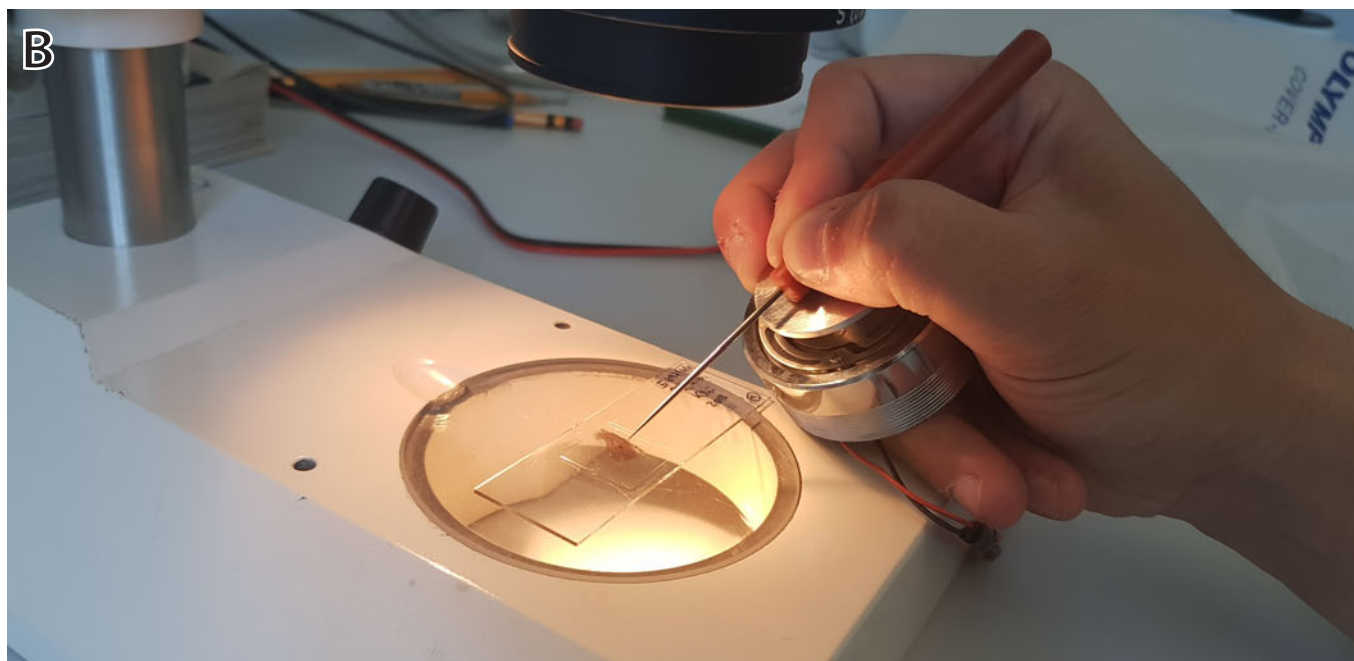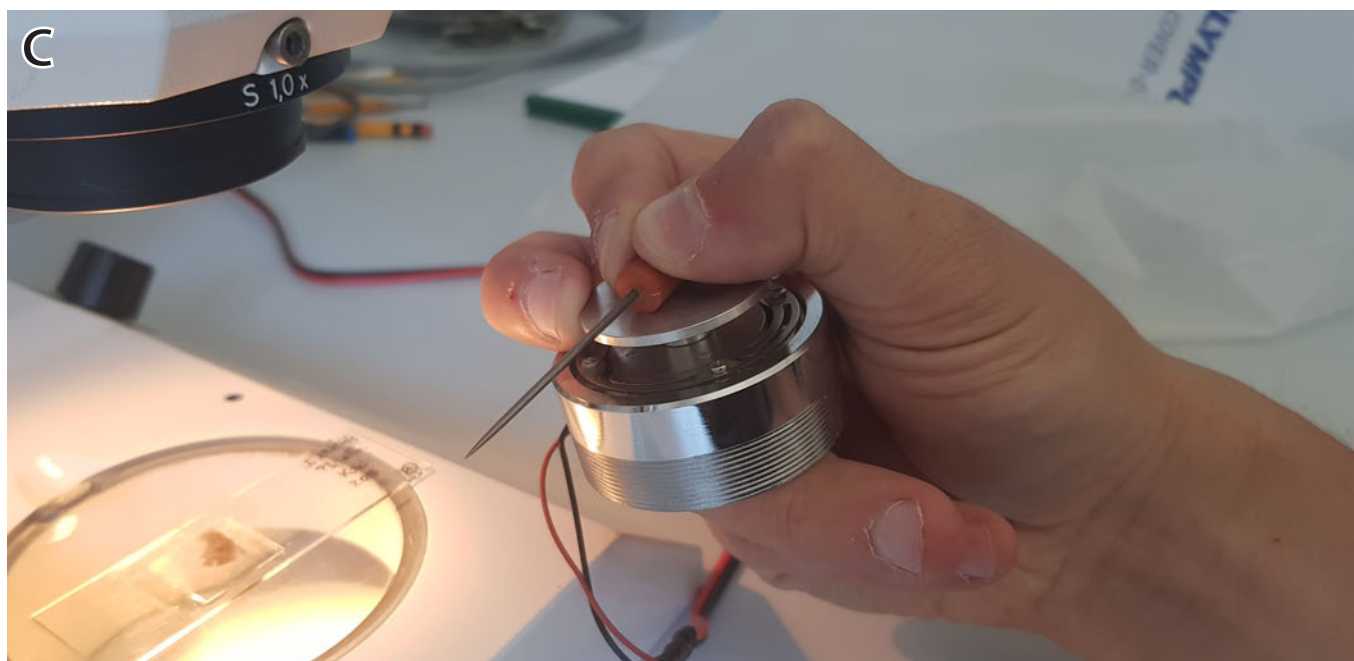

Supplement: Supplemental Information 3 — (A) Fully assembled vibration device with amplifier, resonance speaker, 3D-printed clamp and preparation needle, (B) and (C) holding position of the vibration device without the 3D-printed clamp. [file peerj-09-12471-s003.pdf]

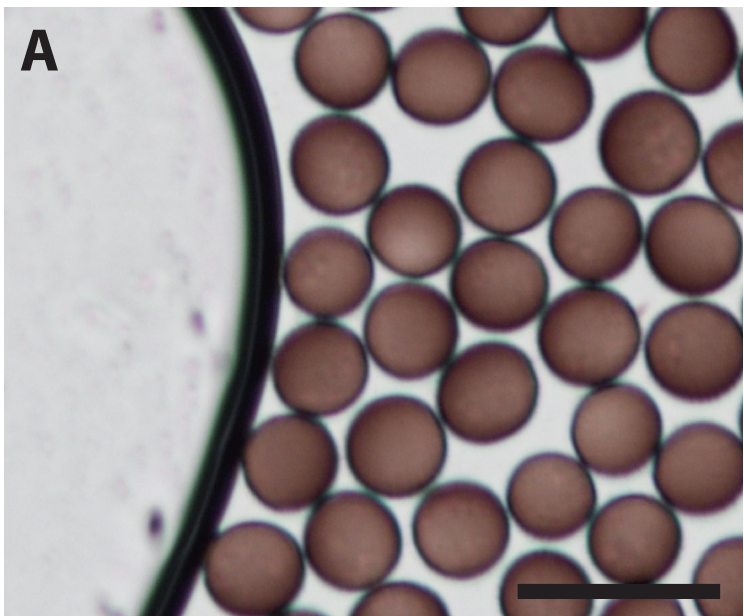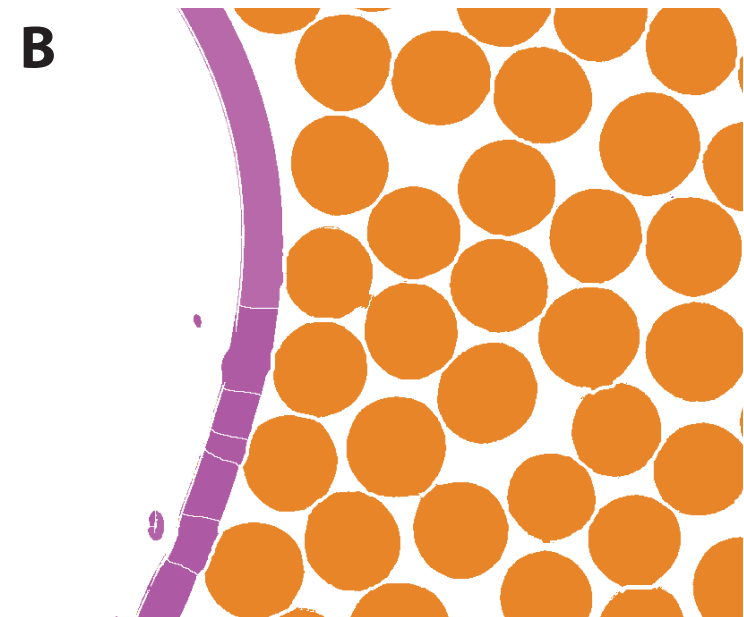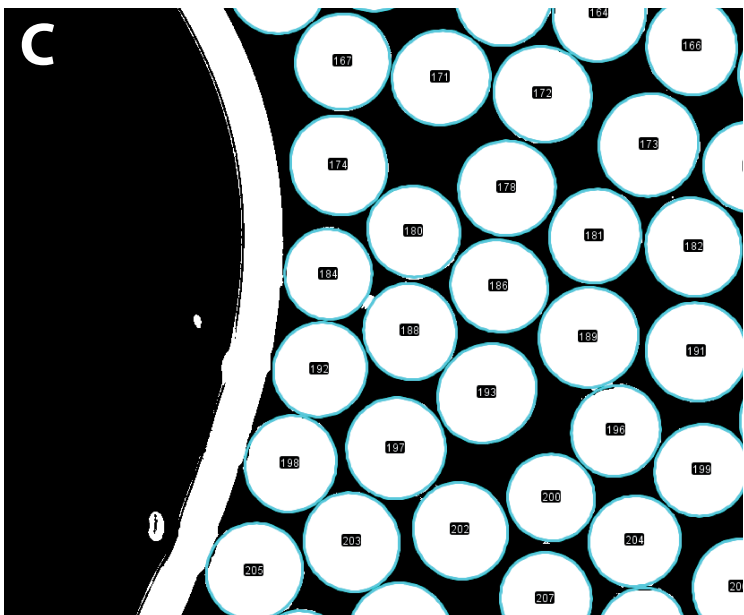

Supplement: Supplemental Information 4 — (A) Original image, bar = 25 µm. (B) Segmented image, recognized target objects in yellow. (C) Target objects labeled. Note the absence of labels for the air bubble and included small amorphous particles. [file peerj-09-12471-s004.pdf]
